# Millipedes faced with drought: the life cycle of a Mediterranean population of *Ommatoiulus sabulosus* (Linnaeus) (Diplopoda, Julida, Julidae)

**DOI:** 10.3897/zookeys.510.8838

**Published:** 2015-06-30

**Authors:** Jean-François David, Mathieu Coulis

**Affiliations:** 1 Centre d’Ecologie Fonctionnelle & Evolutive, UMR 5175, CNRS–Université de Montpellier, 1919 route de Mende, F–34170 Montpellier cedex 5, France

**Keywords:** Millipedes, life cycle, phenology, climate change

## Abstract

Growth, development and life-cycle duration of the millipede Ommatoiulus sabulosus (f. aimatopodus) were studied in a Mediterranean shrubland of southern France and compared with previous data from northwest Europe. Changes in the proportions of stadia during the course of the year were analysed in several generations. The results show that stadia VII and VIII are consistently reached after the first year of growth, and stadia IX and X after the second year. First reproduction may occur at the age of two years in males reaching maturity at stadium X, but not until the age of three in those reaching maturity at stadia XI and XII. Reproduction cannot occur until at least the age of three in females, which carry mature eggs from stadium XI onwards. In comparison with more northern populations, life-cycle duration is not shorter in the Mediterranean population but there are marked differences in its phenology: the breeding period is in autumn, so that juveniles of stadia II to VI are never faced with the summer drought, and larger individuals are mostly inactive in summer; moreover, all individuals moult once every winter. The results illustrate how julid millipedes of humid temperate regions could respond to higher temperatures and drier summer conditions in the context of climate change.

## Introduction

In many organisms, ongoing climate change affects the timing of life-cycle events such as activity, growth and reproduction (Parmesan 2006). When no long-term data sets are available to analyse trends in local populations, potential phenological responses to climate change can be studied by examining intraspecific variation in widespread species that live in a wide range of conditions.

In millipedes (Diplopoda), geographic variation in life-cycle characteristics has been documented for some species of European julids (Fairhurst 1974, David 1982), but there is little precise information for populations living in the Mediterranean region. A number of widespread species such as *Cylindroiulus caeruleocinctus* (Wood), *Cylindroiulus punctatus* (Leach), *Leptoiulus belgicus* (Latzel), *Ommatoiulus rutilans* (C. L. Koch) and *Ommatoiulus sabulosus* (Linnaeus) have populations in this area, which is typified by cool winters and hot, dry summers. The study of phenological characteristics in Mediterranean populations of these species is particularly interesting, because climate change scenarios predict warmer and drier summer conditions over large parts of western Europe for the end of the 21^st^ century (IPCC 2013). The purpose of the present study is to describe the life cycle of a Mediterranean population of *Ommatoiulus sabulosus* and to compare the results with those previously obtained further north in Europe, so as to highlight differences between populations from the two climatic zones. The species has a wide distribution, from Finland and Scotland to Albania and Spain, in contrast to most *Ommatoiulus* species that are confined to the Iberian peninsula and north Africa (Akkari and Enghoff 2012). Two forms occur in southern France: that in which adults have two orange-yellow bands on the back, and a typically Mediterranean form, the so-called *Ommatoiulus sabulosus aimatopodus* (Risso), in which adults are black dorsally. The latter form is the most common in southern France and often occurs at high population densities in shrubland ecosystems on limestone (Coulis et al. 2013).

The post-embryonic growth and development of *Ommatoiulus sabulosus* were described in detail by Halkka (1958) and Sahli (1969). The life cycle, i.e. the calendar of events between birth and reproduction, was studied under field conditions by Halkka (1958) in Finland, Sahli (1968) in Germany, Biernaux (1972) in Belgium and Fairhurst (1974) in Great Britain. Reproduction occurs in late spring and summer in all these regions. Biernaux (1972) tentatively suggested that both males and females mature in two years in Belgium, but Fairhurst (1974) concluded that males take two or three years, and females three or four years, to reach maturity in Great Britain. As regards Mediterranean populations, Sahli (1991a, 1992) studied in detail the timing of male maturation and adult–intercalary male successions (periodomorphosis) in *Ommatoiulus sabulosus aimatopodus* from the Alpes-Maritimes and Provence, southern France. This author mentioned that egg-laying occurs in late summer–early autumn (Sahli 1991a), but provided very limited information on the growth, development and age at reproduction of females. Sahli (1991b) suggested that females could breed only once before dying in Mediterranean populations (semelparity), in contrast to Biernaux (1972) who concluded, based on his own study of egg development in populations from Belgium, that females can breed in successive years (iteroparity). The presence of an abundant population of *Ommatoiulus sabulosus aimatopodus* in a garrigue ecosystem of Provence provided the opportunity to clarify some aspects of the species’ biology in the field, with particular attention to how this julid adjusts its phenology under warmer and drier conditions.

## Methods

This study was conducted at the Massif de l’Etoile near Marseille, southern France (5°25'E; 43°22'N), in a shrubland dominated by rockrose (*Cistus albidus* L.), kermes oak (*Quercus coccifera* L.), rosemary (*Rosmarinus officinalis* L.) and gorse (*Ulex parviflorus* Pourr.). The soil is shallow rendzina on limestone, in which rock fragments and stones represent about 60% of the soil volume in the top 20 cm. The mean annual temperature in the area is 15.1 °C, mean monthly temperatures ranging from 7.1 °C in January to 24.1 °C in July, and the mean annual rainfall is 555 mm (Marseille 1981–2010 climate normals). The driest months are June, July and August, during which the soil becomes very dry. The millipede community, heavily dominated by *Ommatoiulus sabulosus aimatopodus*, also comprises an abundant population of *Polyxenus lagurus* (Linnaeus) (Polyxenidae) and rare specimens of *Leptoiulus* sp. (Julidae) and *Trichoblaniulus* sp. (Trichoblaniulidae).

Collections of millipedes were made using different methods. (1) Twenty three pitfall traps were set on the site in late March 2010 (8 days) and late April 2010 (10 days). (2) Leaf litter and topsoil samples were taken within 25 × 25 cm quadrats in May 2010 (31 sampling units), November 2010 (12 s.u.), May 2012 (31 s.u.), October 2013 (15 s.u.), November 2013 (11 s.u.), March 2014 (12 s.u.), April 2014 (31 s.u.) and September 2014 (13 s.u.). Millipedes were extracted using Tullgren funnels. (3) Large individuals were also collected by hand in leaf litter to determine their reproductive status.

Individuals were assigned to a stadium by counting the rows of ocelli (R.O.) on each side of the head (1 R.O. = stadium II, 2 R.O. = stadium III, etc.) (Enghoff et al. 1993). The method, however, was often difficult to apply from stadium XI onwards. The numbers of podous rings (including the collum) and apodous rings (excluding the telson) were counted. Intercalary males were distinguished from other males by a much smaller first pair of legs than in immature males, but not modified into hooks as in copulatory males (Halkka 1958). Forty-two females of stadia X and higher were dissected to determine whether mature eggs (i.e. brownish, subspherical eggs about 0.6 mm long) were present in the ovitube.

The growth of several cohorts in the field was studied by examining changes in the proportions of stadia in successive samples (Blower 1970). In addition, 40 individuals of various stadia were reared in the laboratory for periods ranging from a few months to two years. They were kept in transparent plastic boxes containing sieved soil and moist leaf litter. The boxes were placed in incubators fitted with a glass door, in which temperature followed the long-term monthly mean temperatures of Marseille, with a daily thermoperiod of low amplitude. All millipedes received a pinch of powder yeast every month and, occasionally, rabbit faeces as supplementary food.

## Results

### Post-embryonic growth and development in the field

The stadia identified using the numbers of R.O., and the numbers of body rings counted in each stadium, are indicated in Table 1. In terms of ring numbers, growth was slightly different from that reported in more northern populations, with an extra apodous ring in stadia III and IV. This was confirmed by the higher numbers of podous rings from stadium IV onwards. The maximum number of stadia is uncertain because it was generally impossible to decipher the exact number of R.O. in the largest individuals. However, a few males had at least 12 R.O. (stadium XIII) and a female with 57 podous rings had at least 13 R.O. (stadium XIV).

**Table 1.** Growth and development of *Ommatoiulus sabulosus* in Provence. The number of rows of ocelli (R.O.), the range of podous rings (collum included) and the numbers of apodous rings (telson excluded) are given for each stadium. Male stages: Im. = Immature; Ad. = adult; Int. = Intercalary.

**Stadium**	**R.O.**	**Podous rings / Apodous rings**			**Male development**
		**Juveniles**	**Females**	**Males**	
II	1	6 / 5			
III	2	11 / 5,6			
IV	3	16–17 / 6,7			
V	4	22–24 / 6,7			
VI	5		29–32 / 6,7,8	29–32 / 6,7	Im.
VII	6		35–38 / 5,6	36–39 / 5,6	Im.
VIII	7		42–45 / 3,4	41–45 / 2,3,4	Im.
IX	8		45–49 / 1,2,3	44–49 / 1,2,3	Im.
X	9		47–50 / 1,2	47–50 / 1,2	Im., Ad.
XI	10		48–53 / 1	48–50 / 1	Im., Ad., Int.
XII+	≥ 11		49–57 / 0,1	50–55 / 0,1	Ad., Int.

Sexual dimorphism was apparent at stadium VI. Although two males reared in the laboratory reached maturity at stadium IX, the smallest adult males found in the field were in stadium X (Table 1). Immature males were numerous up to and including stadium XI, indicating that many males mature for the first time in stadia XI or XII. Intercalary males were found from stadium XI onwards. Dissection of females in late summer–early autumn, just before the breeding period (see below), showed that ovigerous females carrying mature eggs were present in any stadium from stadium XI onwards. None of the stadium X females that were dissected (n = 6) were ovigerous.

### Phenology

**Figure 1.**
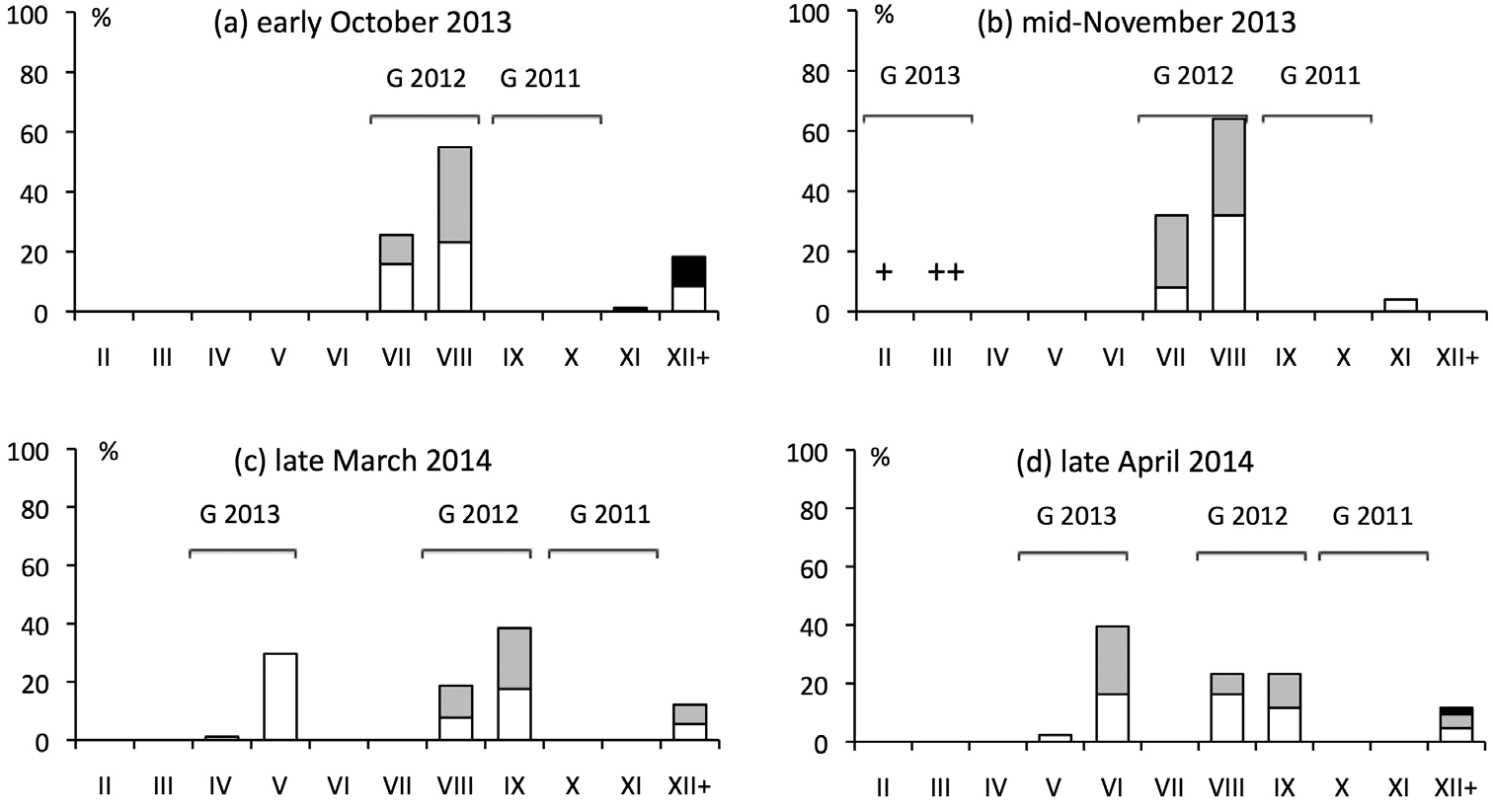
Phenology of *Ommatoiulus sabulosus* in Provence (October 2013–April 2014). Stadia are indicated on the horizontal axis and those of three identifiable generations (G 2011 without any individuals, G 2012 and G 2013) are grouped together. White bars = undifferentiated juveniles and females; grey bars = immature and intercalary males; black bars = adult males. Abundant (+) or very abundant (++) juveniles of stadia II and III were not included in the calculation of percentages.

**Figure 2.**
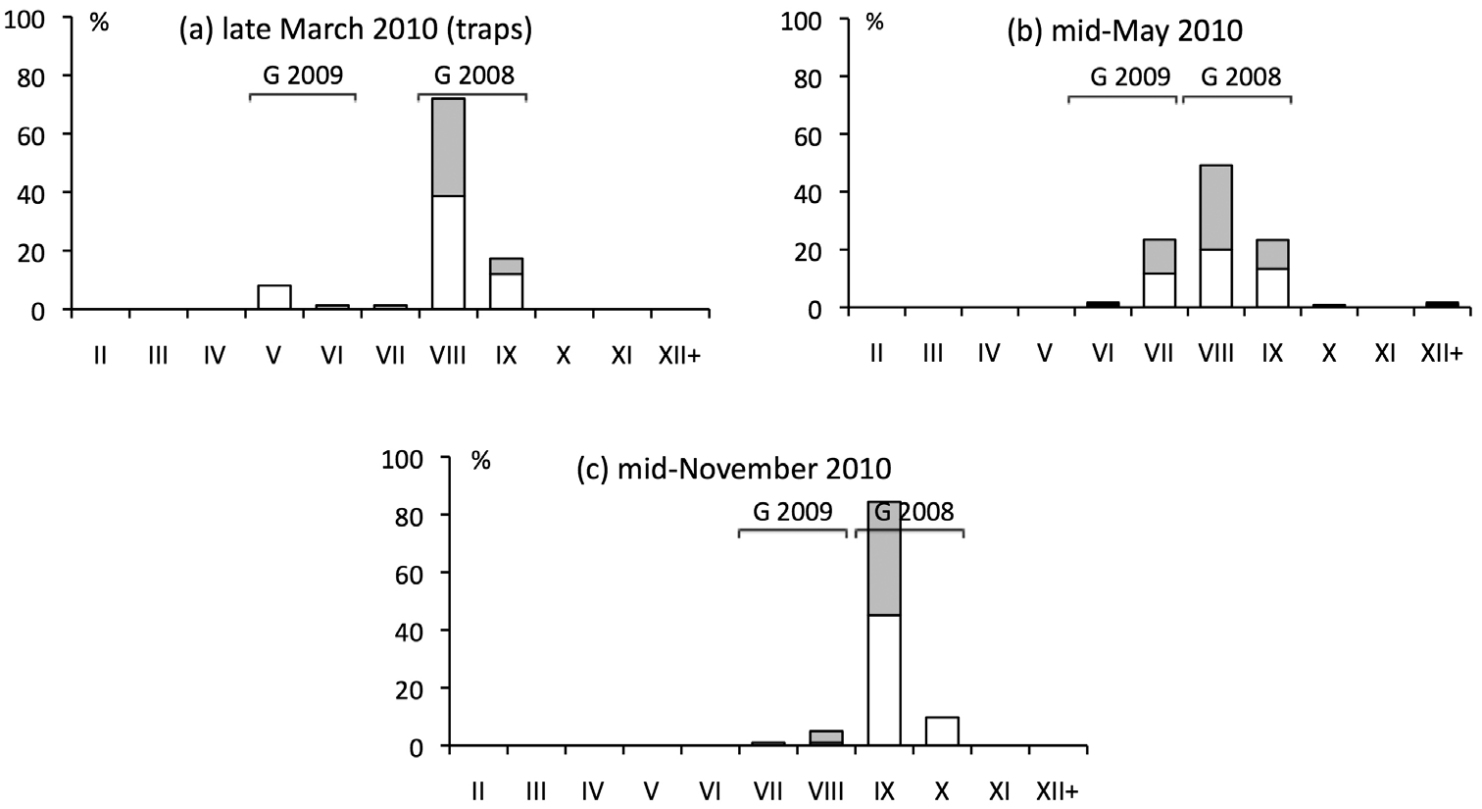
Phenology of *Ommatoiulus sabulosus* in Provence (March 2010–November 2010). See explanations in the legend to Fig. 1.

Juveniles were active in leaf litter in late October as stadium II, in mid-November as stadia II and III (Fig. 1b), and in mid-December as stadia III and IV. Also, a female kept for months in the laboratory produced stadium II juveniles in late October. Samples taken in late March showed that the new generation was mainly in stadium V by the end of winter (Figs 1c, 2a), which implies moulting during the winter. This result was confirmed in laboratory rearings, in which juveniles that had hatched in October emerged from the soil as stadium V in March. The rearings further showed that the new generation continued to grow rapidly in spring, from stadium V in March (rearing temperature: 10 ± 2 °C) to stadium VI in April (13 ± 2 °C) and to stadium VII in May (17 ± 2 °C), exactly as in the field (Fig. 2b). Sexual differentiation at stadium VI thus occurs at the age of about 6 months. The pace of growth slowed markedly around the summer. Litter and topsoil samples taken in early October showed that the smallest individuals, born in autumn of the preceding year, were in stadia VII and VIII (Fig. 1a), indicating that only one moult had occurred since May. All those one-year old millipedes were immature in both sexes.

During the second year of growth, no moult occurred from October to mid-November (Fig. 1b). At this time of year, the population becomes progressively inactive in the soil, both in the field and in the laboratory. One-year old millipedes moulted once during the winter and emerged from the soil in late March as stadia VIII and IX (Figs 1c, 2a). This was confirmed in laboratory rearings, in which six males and females of stadium VIII collected in the field in October burrowed into the soil in October–November and emerged as stadium IX in March. Field samples taken in 2010 (Fig. 2) showed that the generation that was in stadia VIII and IX at the end of winter remained in these stadia until May, moulted in mid-May (as shown by the large proportion of millipedes that were moulting at the time of sampling), and was still in stadia IX and X in mid-November, at the age of two. The complete absence of stadia IX and X in the autumn of 2013 (Fig. 1a, b) indirectly confirms that these stadia are reached in two years, since, for unexplained reasons, there was no recruitment in the autumn of 2011. Similarly, samples taken in the autumn of 2010 (Fig. 2c) suggest there was no or little recruitment in that year, which was confirmed in subsequent samples (data not shown).

The number of moults during the third year of growth cannot be deduced from the field data. Assuming that there are two further moults — one in winter and one in spring, as in the second year of growth — most individuals of stadia XI and XII found in the autumn of 2013 (Fig. 1a) would be three years old. However, this would be inconsistent with the lack of juvenile recruitment in 2010, and it is likely that most individuals in stadia XI and XII collected in the autumn of 2013 were actually born before 2010.

### Life-cycle duration

By combining the results on individual development and phenology, one may infer that a small proportion of males that reach maturity at stadium X reproduce at the age of two. However, many males that mature for the first time at stadia XI and XII cannot reproduce until the age of three. There is no evidence that some females breed at the age of two, since no ovigerous females were found in stadia IX and X in late summer. Females need at least three years to reach stadium XI and lay eggs in early autumn.

The continuation of the life cycle was observed in a few adults reared in the laboratory. Three adult males collected in autumn moulted during the winter and emerged from the soil in March as intercalary males. They became mature again after a further moult in spring and remained in the adult stage until the following autumn. Two large females collected in autumn also moulted during the winter but, in contrast to males, they did not moult again in spring or summer. One of these females bred in October, overwintered a second time in the laboratory, and survived until the following September but without moulting. Post-mortem inspection showed that this female had no apodous ring and contained no eggs.

## Discussion

The present study provides the first estimate of life-cycle duration for *Ommatoiulus sabulosus* in southern France. The interpretation of our field data was made easy by the generally high abundance of juveniles and also by gaps between successive generations, possibly due to reproduction failures and/or high juvenile mortality rates in some years. In the population studied near Marseille, stadia VII and VIII are consistently reached after the first year of growth, and stadia IX and X after the second year. This pattern was observed in three generations born between 2008 and 2012, despite some variation from one year to another (e.g. the cohort born in 2008 was mainly in stadium VIII in late March 2010, while that born in 2012 was mainly in stadium IX in late March 2014). Our results differ from those of Sahli (1992), who assumed that stadia X and XI were reached at the age of three in the region of Provence.

As adult males were found from stadium X onwards in our samples, some males may reproduce at the age of two. However, males that reach maturity in stadia XI or XII cannot reproduce until the age of three at the earliest. Also, ovigerous females, which were found from stadium XI onwards in our samples, cannot breed until the age of three at the earliest. Moreover, the presence of some stadium XI females without any mature eggs in early autumn suggests they may start breeding at the age of four. Therefore, the duration of the life cycle, which corresponds to the age of females at first reproduction, is three or possibly four years in this Mediterranean population, i.e. the same as in populations studied by Fairhurst (1974) in Great Britain.

It remains unclear whether each female breeds only once during its lifetime (semelparity) or can breed over several years (iteroparity). Sahli (1991a) assumed that *Ommatoiulus sabulosus* females might be semelparous in Mediterranean populations, reproduction being spread over different stadia and different years in each generation. In the present study, the single female that bred in the laboratory survived for a further year but died without breeding again, so that there is still no direct evidence for iteroparity. Moreover, we did not find clusters of small oocytes at the same time as mature eggs in ovigerous females, which Biernaux (1972) mentioned as evidence for iteroparity in *Ommatoiulus sabulosus*. On the other hand, dissection of females in late summer–early autumn revealed that the proportion of those not carrying mature eggs in stadia XI and higher was rather low (22%), and the question is whether this is sufficiently high to be consistent with semelparity. Semelparity would imply that many females in stadia XI, XII and even XIII postpone reproduction until the next year(s), which should result in a substantial proportion of females without eggs in early autumn. This topic requires further research.

Although Mediterranean conditions do not modify the length of the life cycle in *Ommatoiulus sabulosus*, several phenological characteristics are very different between the population of Marseille and more northern populations. First, there is a shift of the breeding season. In northwestern Europe, the species generally breeds in summer (Sahli 1968, Biernaux 1972, Fairhurst 1974). Under milder climate conditions, as on the island of Jersey, the species tends to breed earlier (Fairhurst 1974). However, our study confirms that, in the Mediterranean region, the breeding period of *Ommatoiulus sabulosus* is delayed until the autumn (Halkka 1958, Sahli 1991a). Juveniles of stadia II to IV were collected only in this season. They grow rapidly from autumn to the following spring and the first part of the life cycle is similar to that of *Ommatoiulus moreleti* (Lucas) in southern Portugal, which breeds in late autumn–early winter (Baker 1984). In both species, the earliest active stadia (stadia II to VI) are never faced with the hot and dry conditions of the summer, which may be an adaptation of the Mediterranean populations of *Ommatoiulus*. It should be noted that, in other millipedes, the youngest stadia are by far the least resistant to desiccation (David and Vannier 2001).

The seasonal patterns of activity and growth also differ between the two climatic areas. In northern populations, there is generally a single period of activity and growth from spring to autumn and the species is active in summer (Halkka 1958, Fairhurst 1974). The duration of the active season clearly increases with increasing temperatures in areas where the risk of summer drought is low (cf. Halkka 1958, Fairhurst 1974, Meyer 1985). In the Mediterranean population, however, activity stops during the summer months, and our study has shown that there is at most one moult between May and September. Similarly, in *Ommatoiulus moreleti* living at low altitudes in Madeira, Read (1985) reported that growth slows down during the summer, presumably due to dry conditions. The presence of two long periods of inactivity, in summer as well as winter, largely explains why the life cycle of *Ommatoiulus sabulosus* is not shorter under Mediterranean conditions, as would have been expected for millipedes living in a warmer climate (David and Handa 2010).

## Conclusion

The life cycle of *Ommatoiulus sabulosus* in the Mediterranean region appears to be influenced mainly by the summer drought. The dry season especially impacts phenology, i.e. the timing of activity, growth and reproduction. Contrary to many organisms that breed earlier in spring under warmer conditions, this julid breeds in autumn under Mediterranean conditions, so that juveniles are unlikely to be exposed to severe drought. Moreover, larger stadia become inactive in summer and the total duration of activity over a year is roughly the same as in northern populations. As a result, the life cycle is not shorter in the Mediterranean region than in Great Britain. Although it is too soon to generalize, the life cycle of *Ommatoiulus sabulosus* in southern France is quite similar to that of *Ommatoiulus moreleti* in southern Portugal, suggesting ways in which a number of julids could respond to drier summer conditions in the context of climate change.
